# Identification of *SCN5a* p.C335R Variant in a Large Family with Dilated Cardiomyopathy and Conduction Disease

**DOI:** 10.3390/ijms222312990

**Published:** 2021-11-30

**Authors:** Farbod Sedaghat-Hamedani, Sabine Rebs, Ibrahim El-Battrawy, Safak Chasan, Tobias Krause, Jan Haas, Rujia Zhong, Zhenxing Liao, Qiang Xu, Xiaobo Zhou, Ibrahim Akin, Edgar Zitron, Norbert Frey, Katrin Streckfuss-Bömeke, Elham Kayvanpour

**Affiliations:** 1 Department of Medicine III, Institute for Cardiomyopathies Heidelberg (ICH), University of Heidelberg, 69120 Heidelberg, Germany; Farbod.Sedaghat-Hamedani@med.uni-heidelberg.de (F.S.-H.); Safak.Chasan@med.uni-heidelberg.de (S.C.); ttkrause@me.com (T.K.); jan.haas@med.uni-heidelberg.de (J.H.); edgar.zitron@med.uni-heidelberg.de (E.Z.); Norbert.Frey@med.uni-heidelberg.de (N.F.); 2 DZHK (German Centre for Cardiovascular Research), Heidelberg-Mannheim, 17475 Greifswald, Germany; ibrahim.elbattrawy2006@gmail.com (I.E.-B.); xiaobo.zhou@medma.uni-heidelberg.de (X.Z.); Ibrahim.Akin@medma.uni-heidelberg.de (I.A.); 3 Clinic for Cardiology and Pneumology, Georg-August-University Göttingen, 37073 Göttingen, Germany; sabine.rebs@med.uni-goettingen.de (S.R.); katrin.streckfuss@med.uni-goettingen.de (K.S.-B.); 4 DZHK (German Centre for Cardiovascular Research), 37073 Göttingen, Germany; 5 Institute of Pharmacology and Toxicology, University of Würzburg, 97070 Würzburg, Germany; 6 Department of Medicine, University Medical Centre Mannheim (UMM), 68159 Mannheim, Germany; Rujia.Zhong@medma.uni-heidelberg.de (R.Z.); Zhenxing.Liao@medma.uni-heidelberg.de (Z.L.); Qiang.Xu@medma.uni-heidelberg.de (Q.X.)

**Keywords:** familial DCM, *SCN5a*, conduction disease

## Abstract

Introduction: Familial dilated cardiomyopathy (DCM) is clinically variable and has been associated with mutations in more than 50 genes. Rapid improvements in DNA sequencing have led to the identification of diverse rare variants with unknown significance (VUS), which underlines the importance of functional analyses. In this study, by investigating human-induced pluripotent stem cell-derived cardiomyocytes (iPSC-CMs), we evaluated the pathogenicity of the p.C335R sodium voltage-gated channel alpha subunit 5 (*SCN5a*) variant in a large family with familial DCM and conduction disease. Methods: A four-generation family with autosomal dominant familial DCM was investigated. Next-generation sequencing (NGS) was performed in all 16 family members. Clinical deep phenotyping, including endomyocardial biopsy, was performed. Skin biopsies from two patients and one healthy family member were used to generate human-induced pluripotent stem cells (iPSCs), which were then differentiated into cardiomyocytes. Patch-clamp analysis with *Xenopus* oocytes and iPSC-CMs were performed. Results: A *SCN5a* variant (c.1003T>C; p.C335R) could be detected in all family members with DCM or conduction disease. A novel truncating *TTN* variant (p.Ser24998LysfsTer28) could also be identified in two family members with DCM. Family members with the *SCN5a* variant (p.C335R) showed significantly longer PQ and QRS intervals and lower left ventricular ejection fractions (LV-EF). All four patients who received CRT-D were non-responders. Electrophysiological analysis with *Xenopus* oocytes showed a loss of function in *SCN5a* p.C335R. Na^+^ channel currents were also reduced in iPSC-CMs from DCM patients. Furthermore, iPSC-CM with compound heterozygosity (*SCN5a* p.C335R and *TTNtv*) showed significant dysregulation of sarcomere structures, which may be contributed to the severity of the disease and earlier onset of DCM. Conclusion: The *SCN5a* p.C335R variant is causing a loss of function of peak INa in patients with DCM and cardiac conduction disease. The co-existence of genetic variants in channels and structural genes (e.g., *SCN5a* p.C335R and *TTNtv*) increases the severity of the DCM phenotype.

## 1. Introduction

Dilated cardiomyopathy (DCM), with a prevalence of 1 in 250, is one of the major causes of heart failure and is the most common cause of heart transplantation [1,2]. To date, mutations in more than 50 genes have been reported to be associated with DCM; however, the precise pathomechanisms responsible for the variations in disease susceptibility and phenotype expression, including the risk of heart failure (HF) or sudden cardiac death (SCD), are virtually unknown [1,3]. Familial DCM, with an autosomal dominant inheritance pattern, can be seen in about 20% of the cases [1,4]. Mutations in specific genes such as *LMNA*, *RBM20,* and sodium voltage-gated channel alpha subunit 5 (*SCN5a*) are associated with a higher risk of life-threatening arrhythmias and rapid disease progression so that knowing a patients’ genotype would lead to earlier management [1,5].

Cardiac Na^+^ channels, encoded by the *SCN5a* gene, are essential for initiating heartbeats and maintaining a regular heart rhythm [6]. Till now, numerous rare variants in the *SCN5a* gene have been reported in association with inherited atrial or ventricular arrhythmias, such as long QT syndrome (LQTS), Brugada syndrome (BrS), and catecholaminergic polymorphic ventricular tachycardia (CPVT) [7]. Genetic variations in *SCN5a* are also linked to other structural myocardial diseases, including DCM [3,8]. However, most of these are variants with unknown significance (VUS), which are not functionally analyzed yet, and their pathomechanisms are still unclear.

In the present study, we identified and validated the *SNC5a* p.C335R variant in a large family with familial DCM and conduction disease by investigating iPSC-CMs and *Xenopus* oocytes. Furthermore, we showed that the co-existence of two pathogenic variants (*SCN5a* p.C335R and *TTNtv* p.Ser24998LysfsTer28) could affect the severity of the phenotype.

## 2. Material and Methods

### 2.1. Clinical Evaluation

**Figure 1:**
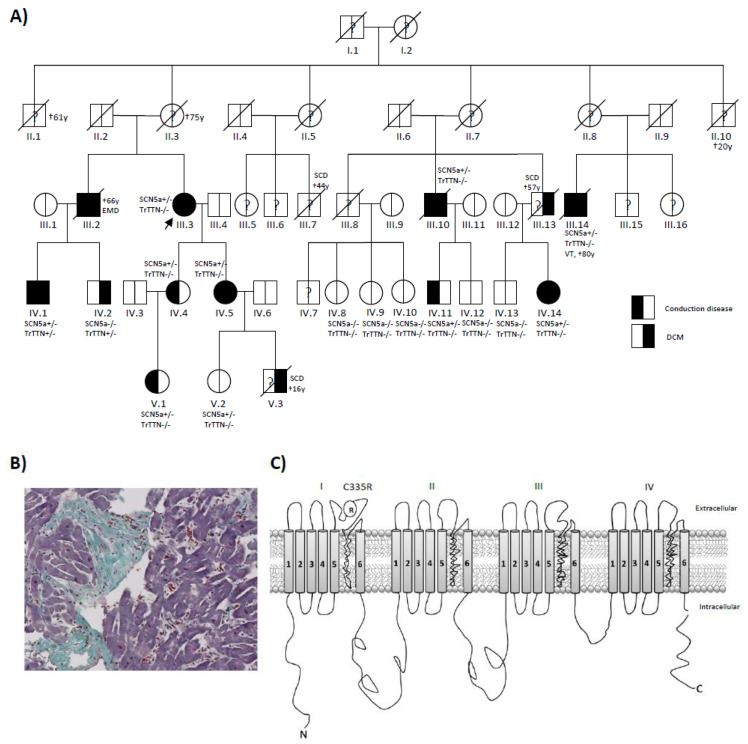
Identifying *SCN5a* p.C335R and *TTN* truncating variants by using precision diagnostic methods. (**A**) Patient’s pedigree showing co-segregation of an *SCN5a* C335R variant. The index patient (III. 3) was diagnosed with DCM and sick sinus syndrome at age 59 and underwent CRT-D implantation. She only carried the *SCN5a* C335R variant. Patient IV.1 was diagnosed with DCM and conduction disease at age 24. He carried both *SCN5a* and *TTN* truncating variants. (**B**) Masson Trichrome stained left ventricular endomyocardial biopsy of patient IV.1 with *SCN5a* and *TTN* truncating variants. Histopathological examination demonstrated extensive cardiac fibrosis in this patient with DCM and conduction disease. (**C**) Schematic representation of the Nav1.5 cardiac sodium channel. The variant is located in Ploop between S5 and S6 of Domain-I.

A four-generation German family with DCM, including 16 family members, was investigated (Figure 1A). Clinical deep phenotyping, including ECGs, echocardiography, cardiovascular magnetic resonance imaging (cMRI), and endomyocardial biopsy, were performed (Figure 1B and Table 1).

### 2.2. DNA Sequencing

DNA Blood Maxi and DNeasy Blood and Tissue kits (Qiagen) were used for DNA isolation. In total, 150 ng of the isolated DNA was used to generate sequencing libraries for Illumina sequencing using Agilents SureSelect Target Enrichment technology. Sequencing was performed on a HiSeq2000 or NovaSeq 6000 (paired-end 2 × 100 bp) in 16 family members. Whole-genome sequencing was performed in iPS-CMs derived from three family members (IV.1, IV.12, and IV.14).

### 2.3. Human Induced Pluripotent Stem Cell-Derived Cardiomyocytes (iPSC-CMs)

Skin fibroblasts of three family members (IV.1, IV.12, and IV.14), who agreed to participate, were isolated from skin biopsies, as described earlier [9]. For plasmid-based integration-free reprogramming, we followed the protocol previously described [9]. For every patient, two iPSC-lines were selected and analyzed for pluripotency. IPSC lines were differentiated into iPSC-CMs by using standardized protocols [9]. Cells were studied 60 days after initiation of differentiation. After differentiation, the purity of hiPSC-CMs was determined by flow cytometry analysis (>90% cardiac TNT+) or by morphology. IPSC-CMs were maintained in RPMI 1640 supplemented with Glutamax, HEPES, and B27 supplement. For sarcomeric regularity in iPSC-CMs, immunofluorescent staining of α-actinin (1:1000, Sigma) and titin-M8/M9 (1:750, MyoMedix) was obtained with a 40× objective. For quantification of regularity, the α-actinin channel was analyzed with fast Fourier transformation (FFT) in ImageJ, and the first-order peak amplitude was calculated.

### 2.4. Site-Directed Mutagenesis and Heterologous Expression in Xenopus Oocytes

*SCN5a* gene, encoding the α-subunit of human Nav1.5 channel, in plasmid pcDNA1.1/Amp was used. The p.C335R variant of *SCN5a* was prepared by introducing a single point mutation by using the QuikChangeTM (Stratagene) site-directed mutagenesis kit and the following oligonucleotides: 5′-ACGCTGGGACACGTCCGGAGGGCTA-3′ (sense) and 5′TAGCCCTCCGGACGTGTCCCAGCGT-3′ (antisense). The mutated *SCN5a* cDNA clone was sequenced to ensure the presence of the p.C335R mutation, as well as the absence of other substitutions introduced by the DNA polymerase. Wild-type and mutant constructs were linearized with XbaI, and cRNAs were synthesized using the mMESSAGE mMACHINE in vitro transcription kit (Ambion, Austin, TX, USA) by the use of T7 Polymerase. Injection of 20–25 ng (46 nl/oocyte) of cRNA into stage V and VI defolliculated *Xenopus laevis* oocytes was performed using a Nanoject automatic injector (Drummond, Broomall, PA, USA). The oocytes were kept in a modified ND96+ solution (100 mM NaCl, 2 mM KCl, 1.8 mM CaCl_2_, 1 mM MgCl_2_, 5 mM HEPES, pH 7.6) at 19 °C. Electrophysiological measurements were made 2 to 4 days after injection at room temperature, and the same solution was used during the measurements.

### 2.5. Electrophysiology Techniques

The two-microelectrode voltage-clamp configuration was used to record currents from *Xenopus laevis* oocytes. Microelectrodes had tip resistances ranging from 0.3 to 0.7 MΩ. Current and voltage electrodes were filled with 3 M KCl solution. The recordings were performed under constant perfusion at room temperature. Currents were elicited by applying a series of voltage clamp steps between −80 mV and +70 mV in 10 mV increments at a pulsing frequency of 1.0 Hz, starting from a holding potential of −120 mV. Data were low-pass filtered at 1 to 2 kHz (−3 dB, four-pole Bessel filter) before digitalization at 5 to 10 kHz. Recordings were performed using a commercially available amplifier (Warner OC-725A, Warner Instruments, Hamden, CT, USA) and pCLAMP software 10.5 (Axon Instruments, Foster City, CA, USA) for data acquisition and analysis.

To measure the peak sodium current (INa) in iPSC-CMs, standard patch-clamp recording techniques were used in the whole-cell configuration. We also used DMZ-Universal Puller (ZeitzInstrumente Vertriebs GmbH, Martinsried, Germany) patch electrodes with a resistance from 1–2.

MΩ were pulled from borosilicate glass capillaries (MTW 150F; world Precision Instruments, Inc., Sarasota, FL, USA) and filled with a pre-filtered pipette solution. Signals, which were acquired at 10 kHz, were filtered at 2 kHz with the Axon 200B amplifier and Digidata 1440A digitizer hardware, as well as pClamp 10.2 software (Molecular Devices, Sunnyvale, CA, USA). Standard patch-clamp recording techniques were used to measure sodium ion channel current peak sodium (I_Na_) at room temperature. The bath solution contained (mmol/L): 20 NaCl, 110 CsCl, 1.8 CaCl_2_, 1 MgCl_2_, 10 HEPES, 10 glucose, 0.001 nifedipine, pH 7.4 (CsOH). Microelectrodes were filled with (mmol/L): 10 NaCl, 135 CsCl, 2 CaCl2, 3 MgATP, 2 TEA-Cl, 5 EGTA, 10 HEPES, and pH 7.2 (CsOH).

### 2.6. Transient Transfection and Western Blotting

CHO cells transfected with cDNA encoding *SCN5A* WT or mutant p.C335R using Viromer Yellow in vitro transfection kit (Lipocalyx GmbH, Halle, Germany) according to the manufacturer’s protocol. Cells were briefly washed in PBS, and then 200 μL of RIPA buffer (50 mM Tris-HCl, 0.5% NP-40, 0.25% NaDeoxycholate, 150 mM NaCl, 1 mM EDTA, 1 mM Na_3_Vo_4_, 1 mM NaF, 0.1% SDS in distilled H_2_O pH 7.4) containing cOmplete^TM^ and Mini Protease Inhibitor Cocktail (Merck KGaA, Darmstadt, Germany) were added to each well. Cells were harvested with the aid of a cell scraper and incubated on ice for 20 min. The resulting extract was cleared of insoluble debris by centrifugation. Protein concentration was determined by using Pierce^TM^ BCA Protein Assay Kit (Thermo Scientific^TM^ Inc., Rockford, IL, USA) according to the manufacturer’s protocol. Proteins were separated via denaturing SDS-PAGE and blotted onto a nitrocellulose membrane. Membranes were blocked in a blocking solution containing 5% (*w*/*v*) skim milk powder and 0.1% Tween 20 in Tris-buffered saline. Primary anti-human NaV1.5 antibodies (Alomone Labs, Jerusalem, Israel) and anti-Rad50 as a reference (Bethyl Laboratories Inc., Montgomery, AL, USA) were diluted in the same blocking solution and incubated with the membranes overnight. Membranes were washed in Tris-buffered saline and then incubated with Donkey anti-Rabbit IgG, HRP-conjugated secondary antibody (GE Healthcare Life Sciences, Buckinghamshire, UK), and diluted in the same blocking solution (1:5000) for 1 h at room temperature. Membranes were washed in Tris-buffered saline. Labeled bands were visualized using Amersham ECL Prime Western Blotting Detection Reagent (GE Healthcare Life Sciences, Buckinghamshire, UK).

### 2.7. Statistics

Data are provided as mean ± standard deviation (SD). Student’s *t*-test was used to analyze the significance between the two groups. A *p*-value < 0.05 was considered statistically significant. In the case of iPSC-CMs, data are presented as mean ± standard error of the mean (SEM) and *p* < 0.05 by one-way ANOVA.

## 3. Results

### 3.1. Clinical Investigation of a DCM Pedigree

**Figure 2:**
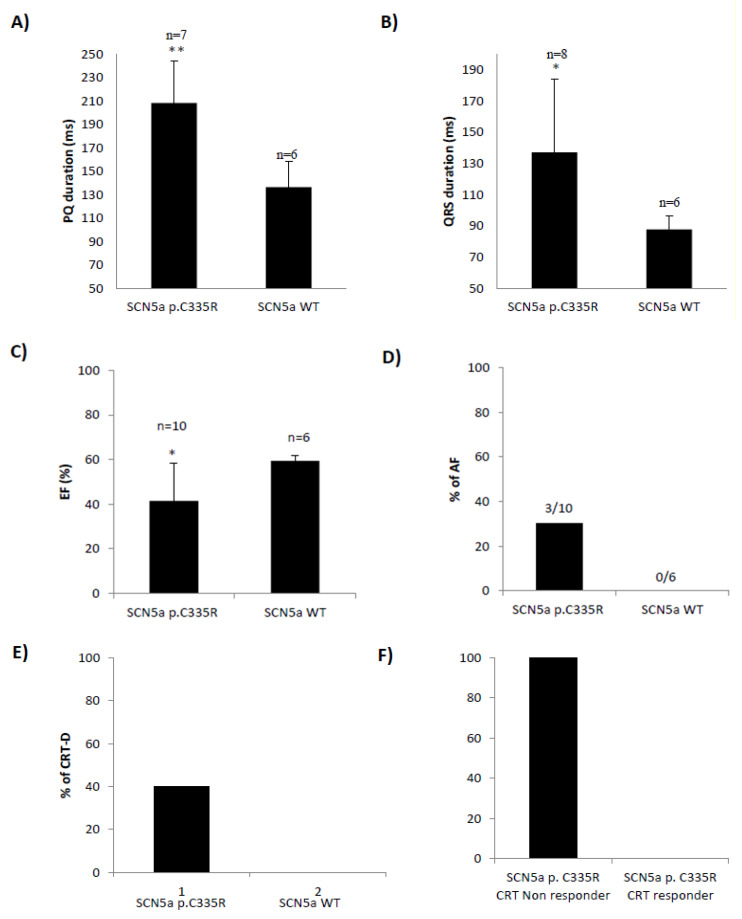
*SCN5a* p.C335R carriers show conduction disease and reduced left ventricular ejection fraction (LV-EF). All family members with *SCN5a* p.C335R variant showed significantly longer PQ (**A**) and QRS intervals (**B**), lower left ventricular EF (**C**), and higher atrial fibrillation (AF) rates (**D**) than other family members without this variant. All 4 family members carrying *SCN5a* p.C335R who received CRT-D were non-responders (**E**,**F**). T-Test; * = *p* ≤ 0.05, ** = *p* ≤ 0.01. Data are presented as mean ± SD.

The index patient (III.3) was diagnosed with DCM and conduction disease at the age of 59 (Figure 1A). Due to symptomatic familial DCM (NYHA III) and complete left bundle branch block (LBBB), the patient received cardiac resynchronization therapy (CRT-D). However, she turned out to be a non-responder. After deep phenotyping, genetic diagnostic testing with next-generation sequencing (NGS) was performed in the index patient and her 15 other family members. Two suspected heterozygous variants in *SCN5a,* and *TTN* could be detected in this family (Supplemental Table S1). In population controls (gnomADv3.1.2), both the *TTN* and *SCN5a* variants have not been detected so far. However, the *SCN5a* variant has been described in Clinvar (NM_000335.5(SCN5A):c.1003T>C, p.Cys335Arg) in a patient with Brugada Syndrome and in one family with atrial fibrillation [10,11]. The *SCN5a* variant is a point mutation (1003T>C) that alters the amino acid at codon 335 from the cysteine (C) to arginine (R) located in the first transmembrane domain (Figure 1C). The second variant was a novel truncating *TTN* variant (NM_001267550.2: c.74987_74991dup) located in the TTN A-band and is constitutively expressed in different isoforms of TTN. This leads to a frameshift with a new termination site (p.Ser24998LysfsTer28). The *TTNtv* variant could only be detected in two other family members with DCM (IV.1 and IV.2) and was not detectable in healthy family members. 1003T>C in *SCN5a* could be detected in all patients with DCM and conduction disease. One patient (IV.2) with DCM but without conduction disease did not have this variant but did have the *TTNtv*. The till-then asymptomatic brother (IV.1) tested positive for both *SCN5a* and *TTNtv*. He developed AV Block III° during an exercise test by routine cardiac evaluation at age 24 and was subsequently diagnosed with DCM and concomitant conduction disease (Supplemental Figure S1). Endomyocardial biopsies of this patient showed severe myocardial fibrosis without evidence for myocarditis (Figure 1B). Family members with *SCN5a* p.C335R showed significantly longer PQ (Figure 2A) and QRS intervals (Figure 2B) and lower LV-EF (Figure 2C). Four of these family members carrying *SCN5a* p.C335R received CRT-D; however, all of them were non-responders (Figure 2E,F and Supplemental Table S2).

### 3.2. The SCN5a p.C335R Variant Is Causing a Loss-of-Function of Sodium Channel Current

**Figure 3:**
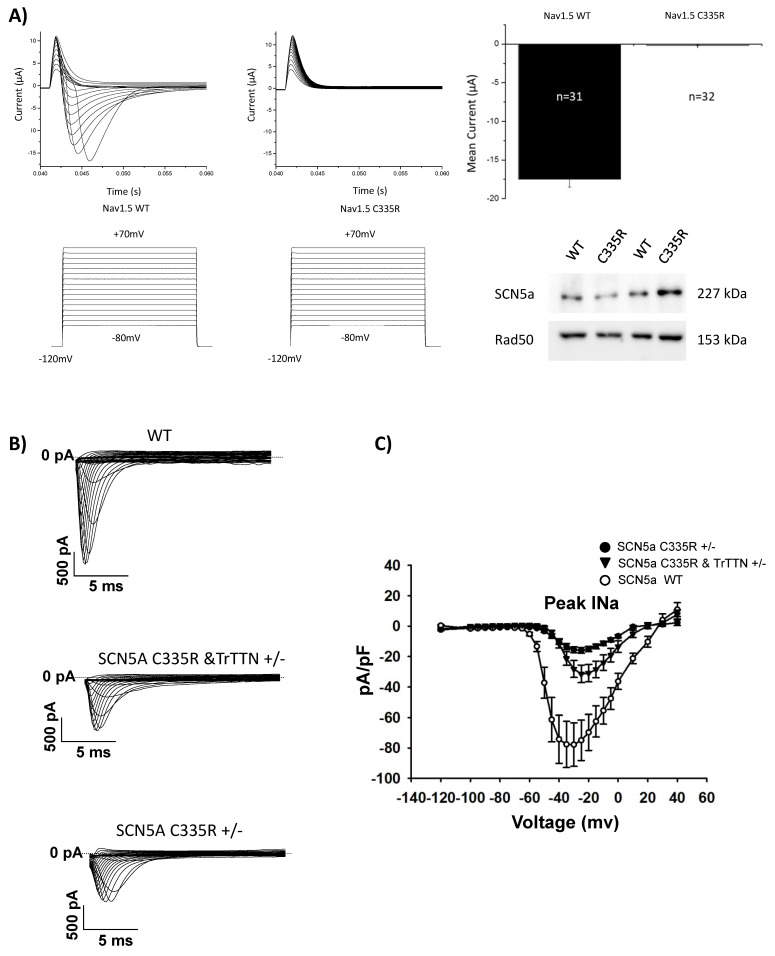
Functional analysis of *SCN5a* p.C335R variant. (**A**) Na^+^ current traces from Nav1.5 wild-type and p.C335R transfected *Xenopus* oocytes. This showed a loss of function in Nav1.5 p. C335R. Sodium channel currents (INa) were evoked from −80 mV to 70 mV. Western blot analysis confirmed that the wild-type and mutated Nav1.5 were expressed and that loss of Nav1.5 function was not due to absent expression of the protein. Anti-Rad50 used as a reference. (**B**,**C**) Representative traces of INa in iPSC-CMs with wild-type and *SCN5a* p.C335R variant. Sodium channel currents (n = 9) were reduced in iPSC-CMs of the DCM patients.

**Figure 4:**
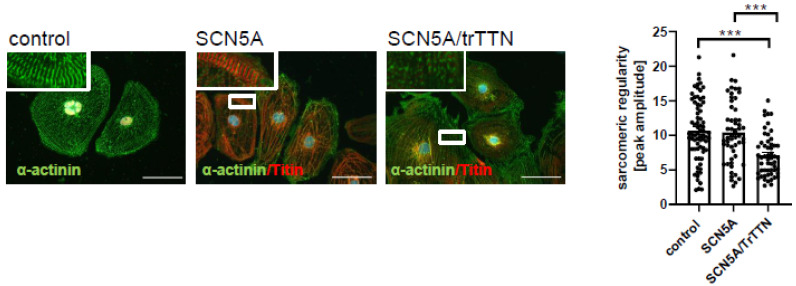
Disturbed sarcomeric z-disc regularity in iPSC-CMs with TTNtv. Sarcomeric structure is visualized by immunofluorescence staining against α-actinin (green) and TitinM8/M9 (red). Scale bar = 50 μm. Using fast Fourier transformation (FFT), the sarcomeric pattern regularity of the α-actinin channel was analyzed. Four cardiac differentiations for control (n = 72) and 3 cardiac differentiations for each patient-specific iPSC line (each n = 54) were analyzed. Data are presented as mean ± SEM. *p* < 0.001 by one-way ANOVA with Tukey’s correction. *** = *p* ≤ 0.001.

p.C335R is located in a highly conserved P-loop (pore-forming region) between S5 and S6 of Domain I of *SCN5a* (Figure 1C). Electrophysiological analysis in *Xenopus* oocytes demonstrated that the *SCN5a* p.C335R variant completely abolished the activity of the human Nav1.5 channel indicating a loss of function of the channel due to the mutated variant (Figure 3A). The *SCN5a* expression in the p.335R variant was assessed at similar levels in comparison to the wild-type by using Western blot analysis, supporting the findings that the mutant caused a loss of function of the channel rather than affecting the expression of the protein (Figure 3A). For further specific analyses, iPSC-CMs were generated from a patient with only *SCN5a* p.C335R (IV.14), another patient with both *SCN5a* p.C335R and *TTNtv* (IV.1), as well as a healthy family member with none of these variants (IV.12). All generated iPSCs were tested regarding pluripotency (Supplemental Figure S2) and differentiated into functional beating cardiomyocytes with a purity of over 95% cardiac cTNT positive cells. Similar to sodium current traces from Nav1.5 in transfected *Xenopus* oocytes, peak Na^+^ channel currents were reduced in iPSC-CMs from DCM patients with the *SCN5a* p.C335R variant (Figure 3B), showing that this mutation led to loss-of-function and decreased peak I_Na_ in patients with cardiac conduction disease and DCM in this family. In contrast, only iPSC-CMs with multiple gene mutations (*SCN5a* p.C335R and *TTNtv*) showed a more significant dysregulation of sarcomere structures compared to iPSC-CMs with *SCN5a* p.C335R or healthy controls (Figure 4), which may explain the severity of the disease and earlier onset of DCM in patient IV.1.

## 4. Discussion

Precise diagnostic tools in cardiomyopathies are essential for accurate diagnosis and treatment. DCM develops due to numerous genetic and non-genetic factors. Family screening and precise genetic evaluation of DCM patients can help to identify these at an earlier stage and improve their survival [12]. Due to rapid and cost-effective DNA sequencing, multigene sequencing panels are now in routine clinical use to diagnose genetic disorders [13,14]. This leads to the identification of thousands of variants with unknown significance (VUS). As a result, interpretation of these VUSs or the consequence of genetic testing and genotype-phenotype relationships are the major challenges in clinical application [15]. False pathogenicity evaluation of variants can lead to negative consequences and psychosocial burden for DCM patients and their relatives [13]. This highlights the importance of functional analysis in these rare novel variants.

Familial DCM patients with *SCN5a* mutations present with a progressive disease with conduction abnormalities and fatal arrhythmias [1]. The cardiac Nav1.5 is encoded by the *SCN5a* gene and mediates the fast influx of Na^+^ (I_Na_), which initiates depolarization of the cardiac cells [16]. Mutations in *SCN5a* cause a variety of cardiac phenotypes depending on gain or loss of function in *SCN5a* [7]. While gain of function mutations lead to an increase of INa and thus longQT syndrome, loss of function mutations cause conduction disease, Brugada syndrome, or DCM [17,18,19]. *SCN5a* p.C335R was reported previously in association with arrhythmia; however, its pathogenicity was not well investigated [10,11,20]. In this study, we identified the *SCN5a* p.C335R variant in a very large family with DCM and conduction disease. To support the pathogenicity of this variant, we performed functional analyses. Na^+^ current traces in Nav1.5 p.C335R transfected *Xenopus* oocytes and in iPSC-CMs with *SCN5a* p.C335R variant showed loss of function. The large Nav1.5 protein consisting of 2016 amino acids contains four domains (D1–D4). Each domain contains six trans-membrane segments (S1–S6) [16]. Between S5 and S6 of each domain is the highly-conserved pore-forming region (P-loop). The *SCN5a* p.C335R variant is located in the P-loop between S5 and S6 of Domain-I. The P-loop of each domain provides a 3D-structure with a hole through which Na^+^ can cross the cell membrane [16]. Any amino acid change in the P-loop can disrupt the Nav1.5 function, as C335R leads to its loss of function. The decrease of I_Na_ affects the intracellular sodium homeostasis, which interrupts Ca^2+^ and H^+^ homeostasis due to Na^+^/Ca^2+^ and Na^+^/H^+^ exchanger. These changes can negatively affect the contractile properties of the cardiomyocyte and cause DCM [21]. Furthermore, DCM could develop secondary to long-time conduction disease at a late stage [7]. There are still no options to activate Nav1.5 in case of its loss of function. CRT-D is recommended in symptomatic DCM patients with LV-EF ≤ 35% and LBBB (class IA and IB) [22]. Based on this recommendation, all patients with the *SCN5a* C335R mutation fulfilling these criteria received CRTs; however, none of them were responders. This finding brings up a new hypothesis that CRT-response may be associated with patients’ genetic background. More studies should be performed to evaluate the genetic background in CRT-non-responder DCM patients.

The *SCN5a* p.C335R variant was the main cause of DCM with conduction disease in this family. However, one patient with both *SCN5a* p.C335R and *TTNtv* showed a more severe phenotype. This example demonstrates that the co-existence of more genetic variants can affect the severity of the phenotype. Nav1.5 interaction partner Telethonin could be one reason for the aggregation of phenotype in these patients. Telethonin is known to interact with titin and has been shown to co-precipitate with *SCN5a* and co-localize in cardiomyocytes [23]. *TTNtvs* occur in 14 to 25% of DCM cases, but the clinical presentation varies from benign to severe [24,25,26]. While Herman et al. reported similar outcomes in DCM patients with and without *TTNtvs*, Corden et al. described *TTNtv* as an important risk factor for severe arrhythmia in patients with DCM [25,27]. Recently, male sex and left ventricular systolic dysfunction were identified as two independent predictors for worse outcomes in *TTNtv* carriers [28]. The reason for this phenotype variability is still unclear; however, the co-existence of pathogenic variants in two different genes could be one of the reasons for wide phenotypic and variable clinical expression of the disease by *SCN5a* or *TTNtv* mutation.

## 5. Conclusions

Functional analysis of rare variants with unknown significance is essential. In this study, we showed that *SCN5a* p.C335R leads to loss of function and DCM with conduction disease. DCM patients, who carried this variant and received CRT-D, were non-responders. Patients’ genetic background, especially mutations in sodium channels, may be one reason for this nonresponse. Furthermore, we showed that the co-existence of multiple mutations could induce a more severe phenotype. These all highlight the importance of precise diagnostics for precise therapy.

## Figures and Tables

**Table 1:** Patient characteristics of the family with *SCN5a* p.*C335R variant*.

ID	Age	Gender	DCM	Conduction Disease	*SCN5a*: C335R	TTNtv	Age at Onset	NYHA	EF	Rhythm	Outcome
III.2	66	M	Yes	RBBB	NA	NA	60	II	15	AF, PM	CRT-D, †66y:electromechanical dissociation
III. 3	78	F	Yes	LBBB, SSS	+/−	−/−	59	III	35	AF, PM	CRT-D
III.10	79	M	Yes	LBBB, SSS	+/−	−/−	63	III	30	AF, PM	CRT-D, †79y
III.14	80	M	Yes	LBBB, SSS, AVB-III°	+/−	−/−	64	III	20	AF, PM	VT 70y, CRT-D, ICD therapy 72 and 78y, †80y
IV.1	35	M	Yes	LBBB, AVB-III°	+/−	+/−	24	I	48	SR	CPR 34y AVB-III°, PM
IV.2	33	M	Yes	No	−/−	+/−	33	I	45	SR	
IV.4	55	F	No	AV-I°	+/−	−/−	49	I	55	SR	
IV.5	44	F	Yes	LAH	+/−	−/−	44	I	40	SR	
IV.8	36	F	No	No	−/−	−/−	-	I	60	SR	
IV.9	45	F	No	No	−/−	−/−	-	I	59	SR	
IV.10	44	F	No	No	−/−	−/−	-	I	60	SR	
IV.11	45	M	No	RBBB	+/−	−/−	45	I	56	SR	
IV.12	49	M	No	No	−/−	−/−	-	I	60	SR	
IV.13	64	M	No	No	−/−	−/−	-	I	62	SR	
IV.14	61	F	Yes	LBBB	+/−	−/−	57	I	50	SR	
V.1	19	F	No	AV-I°	+/−	−/−	19	I	59	SR	x2 Syncope 22y
V.2	18	F	No	No	−/−	−/−	-	Î	60	SR	

AF: atrial fibrillation; AVB: atrioventricular block; CRT-D: cardiac resynchronization therapy-defibrillator; DCM: dilated cardiomyopathy; F: female; ICD: implantable cardioverter-defibrillator; LAH: left anterior hemiblock; LBBB: left bundle branch block; M: male; NA: not available; NYHA: New York Heart Association; SR: sinus rhythm; PM: pacemaker; RBBB: right bundle branch block; SSS: sick sinus syndrome; y: year; †: death.

## Supplementary Materials

The following are available online at https://www.mdpi.com/article/10.3390/ijms222312990/s1.
