# Supplementary material for: Identification of SCN5a p.C335R Variant in a Large Family with Dilated Cardiomyopathy and Conduction Disease
Source: Int J Mol Sci. 2021 Nov 30;22(23):12990. doi: 10.3390/ijms222312990 (PMC8657717; doi:10.3390/ijms222312990)

Supplemental Figure Legend

Supplemental Figure S1: Exercise-induced high grade atrioventricular (AV) block in patient with SCN5a C335R and TTNtv (IV.1).

Supplemental Figure S2 : Generated patient-specific induced pluripotent stem cells (iPSC) show full pluripotency characteristics (A-D). Pluripotency characterization is shown exemplary for iPSC clones from patient IV.14. **A**: The iPSC exhibited typical round colony-forming stem cell morphology (left) and were positive for alkaline phosphatase activity (right). Scale bars: 200 µm. **B**: The generated iPSC express pluripotency markers OCT4, SOX2, LIN28 and GDF3 on the mRNA level in similar amounts to an already published iPSC line (PC = positive control). Somatic cells like fibroblasts (FB) and mouse embryonic fibroblasts (MEF) are negative for expression of these pluripotency genes. GAPDH was used as a housekeeping gene. iPSC-IV.14 c1 and iPSC-IV.14 c2 represent iPSC clone 1 and 2 of patient IV.14. **C**: The iPSC cell lines express pluripotency markers on the protein level, which is visualized by immunoflurescent stainings against *OCT4*, *SOX2*, *NANOG*, *LIN28* and *TRA1-60*. Scale bar: 100 µm. D: In vitro embryoid body formation assay confirmed differentiation capacity into all three germlayers. Postive signals were obtained after immunofluorescent staining against endodermal marker AFP, mesodermal marker α-SMA and ectodermal marker β-tubulin (β-TUB). Scale bars: 100 µm.

Supplemental Figure S1

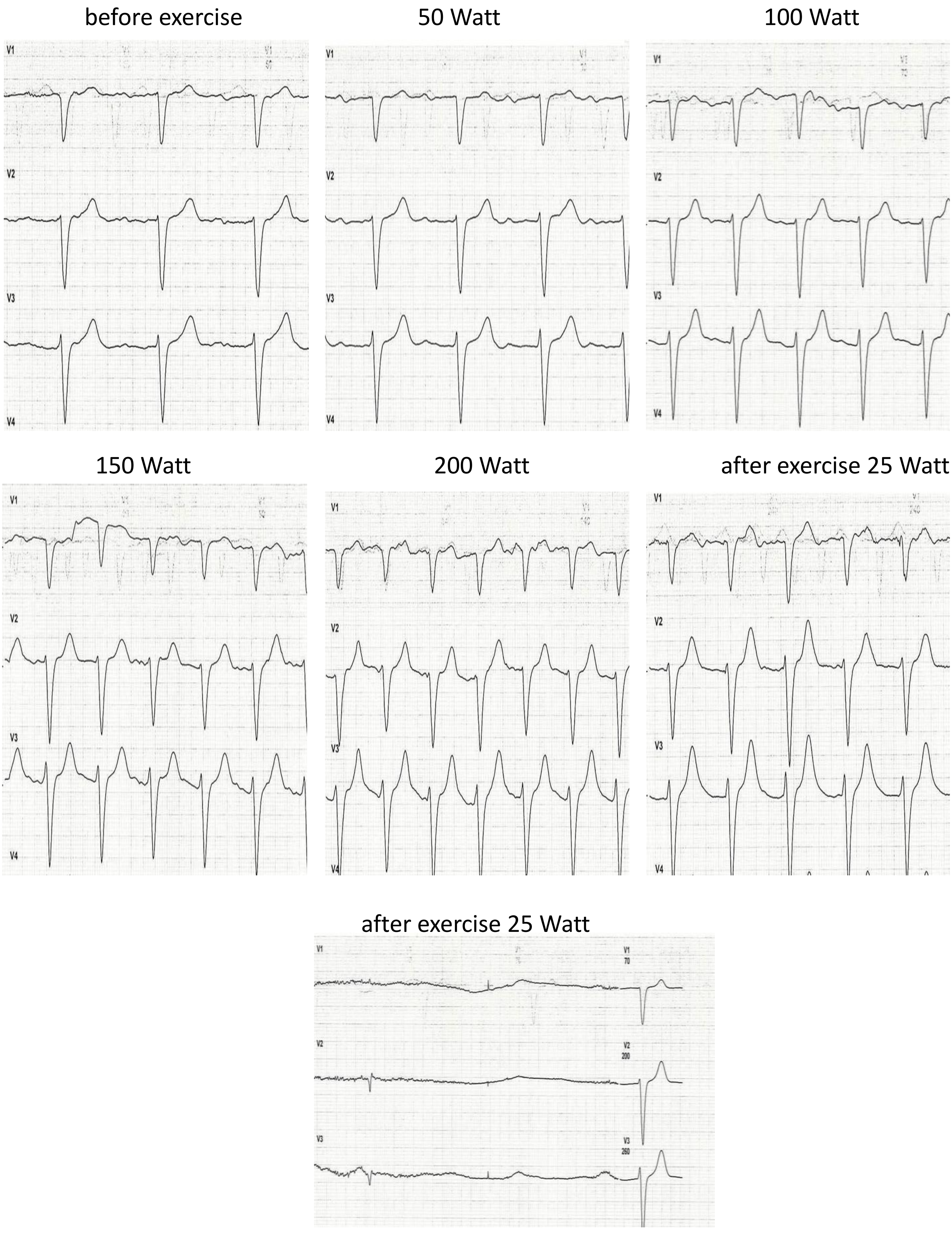

Supplemental Figure S2

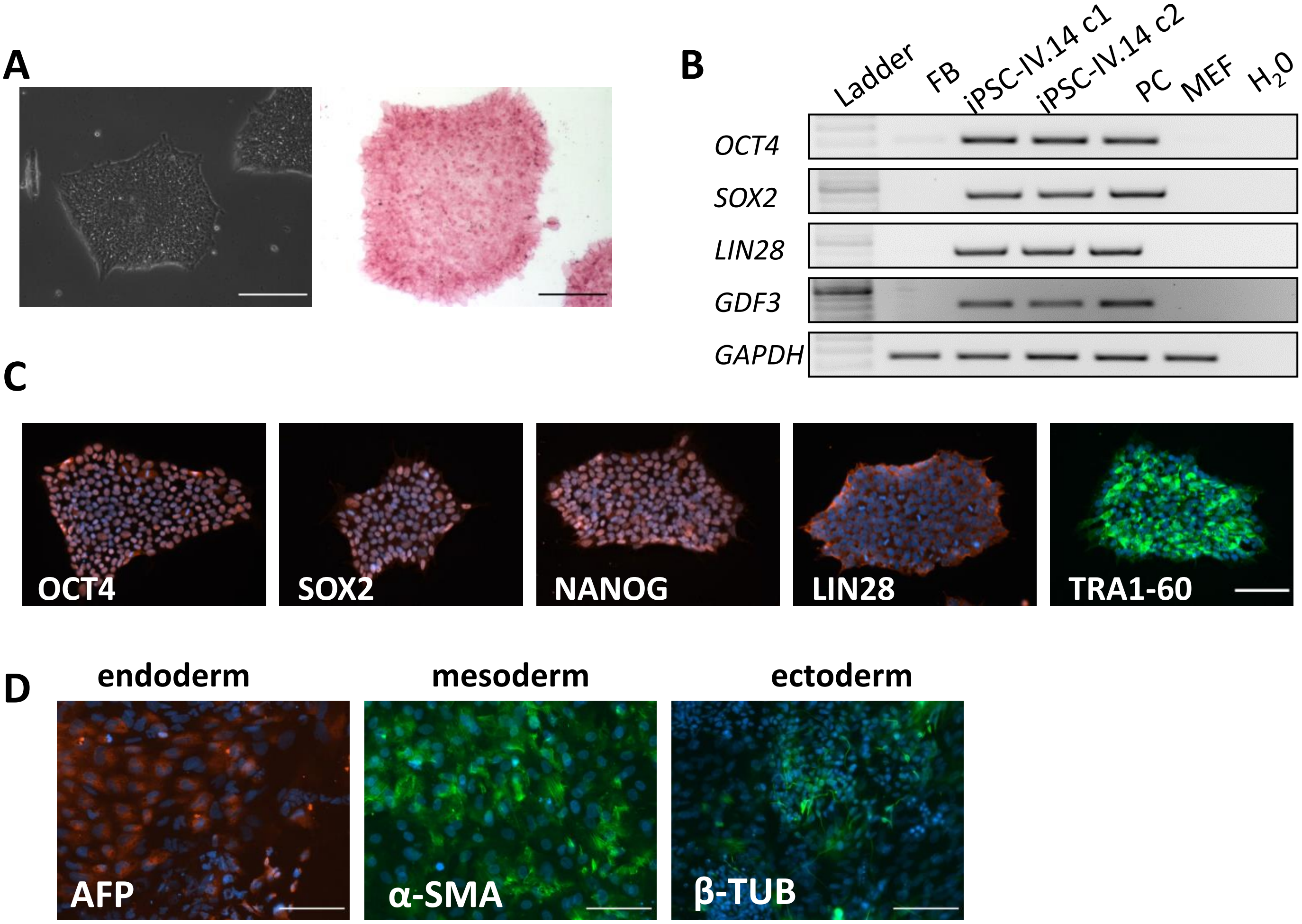

Supplement: Supplementary file 1 [file ijms-22-12990-s001.zip › Supplemental Figure.pdf]
