# Supplementary material for: Identification of SCN5a p.C335R Variant in a Large Family with Dilated Cardiomyopathy and Conduction Disease
Source: Int J Mol Sci. 2021 Nov 30;22(23):12990. doi: 10.3390/ijms222312990 (PMC8657717; doi:10.3390/ijms222312990)
Supplement: Supplementary file 1 [file ijms-22-12990-s001.zip › Supplemental Tables.pdf]

Supplemental Table S1: List of variants

	Gene	Position (GRCh38)	Amino acid change	HGVSc	ACMG classification*
1	SCN5A	chr3:38606806	p.Cys335Arg	SCN5A(NM_198056.3):c.1003T>C	Likely Pathogenic (PM1;PM2;PP2;PP3;PP5)
2	TTN	chr2:178571140	p.Ser24998LysfsTer28	TTN(NM_001267550.2):c.74987_74991dup	Pathogenic (PVS1;PM2;PP5;PP3)

Supplemental Table S2: Patient characteristic before and after CRT implantation

	Before CRT implantation							about 1 year After CRT implantation						
**Patiens**	EF	NYHA	LVEDD	NT-ProBNP	MR	6MWT	QRS	EF	NYHA	LVEDD	NT-ProBNP	MR	6MWT	QRS
**III.2**	NA	II	NA	NA	NA	NA	220	15	III	57	11293	I°	228	228
**III.3**	38	II	57	812	-	441	256	35	II	56	1827	I°	463	223
**III.10**	35	II	55	727	I°	422	212	35	II	55	1825	I°	425	201
**III.14**	NA	II	NA	NA	NA	NA	166	25	II	58	834	I°	NA	195

Supplementary table S3: The list of the genes for panel sequencing.

gene	ENSEMBL ID
**ABCC9**	ENSG00000069431
**ACTC1**	ENSG00000159251
**ACTN2**	ENSG00000077522
**ADRB1**	ENSG00000043591
**ADRB2**	ENSG00000169252
**ADRB3**	ENSG00000188778
**ANKRD1**	ENSG00000148677
**BAG3**	ENSG00000151929
**CACNA1C**	ENSG00000151067

**CACNB2**	ENSG00000165995
**CALR3**	ENSG00000269058
**CASQ2**	ENSG00000118729
**CAV3**	ENSG00000182533
**CBFB**	ENSG00000067955
**CRYAB**	ENSG00000109846
**CSRP3**	ENSG00000129170
**CTF1**	ENSG00000150281
**DES**	ENSG00000175084
**DMD**	ENSG00000198947
**DSC2**	ENSG00000134755
**DSG2**	ENSG00000046604
**DSP**	ENSG00000096696
**EYA4**	ENSG00000112319
**GPD1L**	ENSG00000152642
**ILK**	ENSG00000166333
**JPH2**	ENSG00000149596
**JUNB**	ENSG00000171223
**JUP**	ENSG00000173801
**KCNE1**	ENSG00000180509
**KCNE2**	ENSG00000159197
**KCNH2**	ENSG00000055118
**KCNJ2**	ENSG00000123700
**KCNJ8**	ENSG00000121361
**KCNQ1**	ENSG00000053918
**KCNQ2**	ENSG00000075043
**LAMP2**	ENSG00000005893
**LDB3**	ENSG00000122367
**LIMS1**	ENSG00000169756

**LIMS2**	ENSG00000072163
**LMNA**	ENSG00000160789
**MYBPC3**	ENSG00000134571
**MYH6**	ENSG00000197616
**MYH7**	ENSG00000092054
**MYL2**	ENSG00000111245
**MYL3**	ENSG00000160808
**MYOZ2**	ENSG00000172399
**MYPN**	ENSG00000138347
**NEXN**	ENSG00000162614
**PARVB**	ENSG00000188677
**PDLIM3**	ENSG00000154553
**PKP2**	ENSG00000057294
**PLN**	ENSG00000198523
**PRKAG2**	ENSG00000106617
**PSEN1**	ENSG00000080815
**PSEN2**	ENSG00000143801
**RBFOX1**	ENSG00000078328
**RBM20**	ENSG00000203867
**RYR2**	ENSG00000198626
**SCN1B**	ENSG00000105711
**SCN4B**	ENSG00000177098
**SCN5A**	ENSG00000183873
**SGCD**	ENSG00000170624
**SMYD1**	ENSG00000115593
**SMYD2**	ENSG00000143499
**SNTA1**	ENSG00000101400
**TAZ**	ENSG00000102125
**TBX20**	ENSG00000164532

**TCAP**	ENSG00000173991
**TGFB3**	ENSG00000119699
**TMPO**	ENSG00000120802
**TNNC1**	ENSG00000114854
**TNNI3**	ENSG00000129991
**TNNT2**	ENSG00000118194
**TPM1**	ENSG00000140416
**TTN**	ENSG00000155657
**VCL**	ENSG00000035403
